# Management of Anterior Cruciate Ligament Injuries in Children and Adolescents: A Systematic Review

**DOI:** 10.1186/s40798-025-00844-7

**Published:** 2025-04-23

**Authors:** Hongfu Jin, Nouman Tahir, Shide Jiang, Herasimenka Mikhail, Volotovski Pavel, Masoud Rahmati, Seung Won Lee, Wenfeng Xiao, Yusheng Li

**Affiliations:** 1 Department of Orthopedics, Xiangya Hospital of Central South University, Changsha, 410008 Hunan China; 2 Department of Orthopedics, Loudi Central Hospital, Loudi, Hunan China; 3 Republican Scientific and Practical Center of Traumatology and Orthopedics, Minsk, 220024 Belarus; 4 Department of Physical Education and Sport Sciences, Faculty of Literature and Huma n Sciences, Lorestan University, Khoramabad, Iran; 5 Department of Precision Medicine, Sungkyunkwan University College of Medicine, Suwon, Republic of Korea; 6 National Clinical Research Center for Geriatric Disorders, Xiangya Hospital, Central South University, Changsha, 410008 Hunan China

**Keywords:** Anterior Cruciate Ligament, ACL, Paediatric, Skeletally Immature, Surgery

## Abstract

**Background:**

Due to rising sports participation, anterior cruciate ligament (ACL) tears are increasingly prevalent in children and adolescents. This systematic review aimed to evaluate and summarize the management strategies for ACL injuries in children and adolescents.

**Methods:**

A comprehensive search of PubMed, Embase, Web of Science, and Cochrane Library databases was conducted to identify studies reporting outcomes of ACL injuries in children and adolescents. Key outcomes were synthesized descriptively, including knee instability, secondary damage, growth disturbances, and return-to-sport (RTS) rates.

**Results:**

A total of 7,507 publications were initially screened, with 105 studies involving 8294 children or adolescents satisfying the inclusion criteria. Conservative treatments were associated with elevated rates of knee instability (35.85-100%), secondary meniscal and cartilage damage, and long-term degenerative changes. Conversely, surgical interventions, including physeal-sparing and transphyseal techniques, demonstrated superior outcomes with lower instability rates (0-7.41%), fewer complications, and higher RTS rates (83.4-92.6%). Pooled RTS rates for conservative treatments were 44.0% (95%CI: 0.018–0.927), while physeal-sparing ACL reconstruction showed a pooled RTS rate of 92.6% (95%CI: 0.732-1.000) and transphyseal ACL reconstruction reported an RTS rate of 83.4% (95%CI: 0.722–0.924).

**Conclusion:**

Conservative management of ACL injuries in children and adolescents is linked to higher rates of knee instability, secondary meniscal and cartilage damage, and degenerative changes. In contrast, surgical interventions, such as physeal-sparing and transphyseal techniques, yield better outcomes in knee stability, complications reduction, and RTS rates. However, risks such as graft rupture, repeat surgeries, and potential growth disturbances emphasize the importance of tailoring surgical approaches to the patient’s growth stage and anatomical characteristics.

**Supplementary Information:**

The online version contains supplementary material available at 10.1186/s40798-025-00844-7.

## Key Points

• Conservative treatments for ACL injuries in children and adolescents are associated with higher rates of knee instability, secondary meniscal and cartilage damage, and long-term degenerative changes.

• Surgical interventions, such as physeal-sparing and transphyseal techniques, demonstrate superior outcomes, including improved knee stability, reduced complications, and higher RTS rates.

• Tailored surgical approaches based on growth stage and anatomical characteristics are critical to minimizing risks of graft rupture, repeat surgeries, and potential growth disturbances.

**Supplementary Information:**

The online version contains supplementary material available at 10.1186/s40798-025-00844-7.

## Introduction

As sports participation continues to rise among children and adolescents, anterior cruciate ligament (ACL) injuries have emerged as a notable concern within this active population [1]. These injuries lead to knee instability, pain, and functional impairment, significantly affecting the ability to engage in sports and physical activities [2, 3]. Open physes in children and adolescents present unique challenges for clinicians in managing ACL injuries. The primary concern when operating on skeletally immature children and adolescents is the risk of iatrogenic injury to the developing epiphyses and physes, which can lead to growth disturbances, leg-length discrepancies, angular deformities, and tibial recurvatum [4]. Conservative treatments, such as physical therapy, activity modification, bracing, or splinting/casting of ACL injuries, are considered to protect physes and minimize the risk of growth disturbances [5]. Therefore, surgery was recommended to be postponed until skeletal maturity was reached to avoid compromising growth and development and resulting in growth abnormalities. However, non-surgical treatments, including bracing or restricting physical activity, may cause secondary damage to the meniscus and/or cartilage, impeding the ability of these children and adolescents to return to their previous activity levels [6, 7].

Management strategies for ACL injuries in children and adolescents have evolved considerably over the last few decades. Growing evidence suggests that early ACL reconstruction reduces knee instability and is more likely to restore previous activity levels without affecting the open physes or causing growth disturbances [8]. Multiple reconstructive techniques have been developed, including transphyseal, physeal-sparing, partial transphyseal techniques, and additional augments [9–11]. However, for skeletally immature individuals, it is crucial to note that children and adolescents undergoing ACL reconstruction are at a significantly higher risk of graft rupture compared to adults during long-term follow-up [12]. There are still no guidelines for optimal treatment strategies for managing ACL injuries in children and adolescents. This study aims to systematically evaluate and summarize the current management strategies for ACL injuries in children and adolescents, providing evidence-based guidance to orthopedic surgeons and researchers in optimizing treatment options for this high-risk population. We hypothesize that early ACL reconstruction in skeletally immature children and adolescents leads to better long-term functional outcomes and lower rates of secondary complications compared to conservative management or delayed surgery.

## Methods

### Study Design and Protocol Registration

This study is a systematic review designed to evaluate and summarize the current management strategies for ACL injuries in skeletally immature children and adolescents. This study followed the Preferred Reporting Items for Systematic Reviews and Meta-analysis (PRISMA) reporting guidelines and was preregistered on the PROSPERO database (Registration ID: CRD42023481292).

### Inclusion and Exclusion Criteria

The inclusion criteria for this systematic review were clinical studies published in English-language, peer-reviewed journals, encompassing cohort studies, case-control studies, case series, and cross-sectional studies. Eligible studies had to involve children and/or adolescents with open physes who had sustained ACL injuries. Studies focusing specifically on this population and reporting relevant clinical outcomes, such as return-to-sport (RTS) rates, functional outcomes, and complications (e.g., graft failure, meniscal or cartilage injuries, knee instability, growth disturbances, or leg-length discrepancies), were considered for inclusion. There were no restrictions based on publication year. Exclusion criteria included narrative reviews, systematic reviews, meta-analyses, editorials, conference abstracts, and other non-clinical studies. Furthermore, studies with incomplete data on key clinical outcomes were excluded from the review.

### Data Sources and Search Strategy

A comprehensive literature search was conducted across multiple electronic databases, including PubMed, Embase, Cochrane Library, and Web of Science, covering publications from their inception to October 17, 2023. The search strategy used Medical Subject Headings (MeSH) terms and keywords to capture relevant studies. The terms used included: “Anterior Cruciate Ligament Injuries”, “Anterior Cruciate Ligament Injury”, “Anterior Cruciate Ligament Tear”, “Anterior Cruciate Ligament Rupture”, “ACL Injuries”, “ACL Injury”, “ACL Tear”, “ACL Rupture”, “Child”, “Children”, “Adolescent”, “Adolescence”, “Pediatric”, “Youth”, “Teenager”, “Teen”, and “Skeletally Immature”.

### Study Selection

Two independent researchers (H.J. and N.T.) initially screened the studies based on their study designs and publication categories. Any disagreements during this phase were resolved through discussion or consultation with a third researcher (S.J.). In the second phase, the researchers assessed the eligibility of all studies identified in the initial screening by reviewing their titles and abstracts. Finally, a full-text screening was conducted for the remaining studies to exclude those that lacked original texts, contained incomplete data, or included interventions or populations that did not meet the predefined inclusion criteria.

### Data Extraction

Data were extracted from the included studies using a predefined data extraction form. Two independent researchers (H.J. and N.T.) reviewed all studies to collect information on participant demographics, study design, intervention details, functional outcomes, complications, and other relevant variables. Any disagreements between the researchers were resolved through discussion or consultation with a third researcher (S.J.) to ensure the accuracy and consistency of the extracted data.

### Quality Assessment

The Methodological Index for Nonrandomized Studies (MINORS) was used to evaluate the quality of the included studies. Two independent researchers (H.J. and N.T.) assessed each study’s methodological quality and risk of bias, focusing on aspects such as the selection of study groups, exposure, outcomes, and statistical analysis. Disagreements between the reviewers were resolved through discussion or, if necessary, consultation with a third researcher (S.J.). The quality assessment results were considered during the synthesis and interpretation of the findings, ensuring that the conclusions drawn reflect the reliability and robustness of the included studies.

### Synthesis Methods

The synthesis of the included studies was carried out using descriptive methods. We summarized the study characteristics, including participant demographics, interventions, and key outcomes such as RTS rates, postoperative complications, and functional outcomes. The meta-analysis of single-group proportions was performed to evaluate RTS rates across different interventions for ACL injuries in children and adolescents. The analysis was conducted using the “metaprop” package in STATA software (version 15.1). A random-effects model was applied to account for variability between studies. Heterogeneity among the included studies was assessed using a chi-squared test, and the I² statistic was used to quantify the proportion of total variation attributed to heterogeneity, with I² > 50% indicating substantial heterogeneity. The RTS rates were calculated, and the pooled proportions and their corresponding 95% confidence intervals (CIs) were reported. Forest plots were generated to visualize the pooled results and the variability across studies.

## Results

### Study Selection

**Fig. 1:**
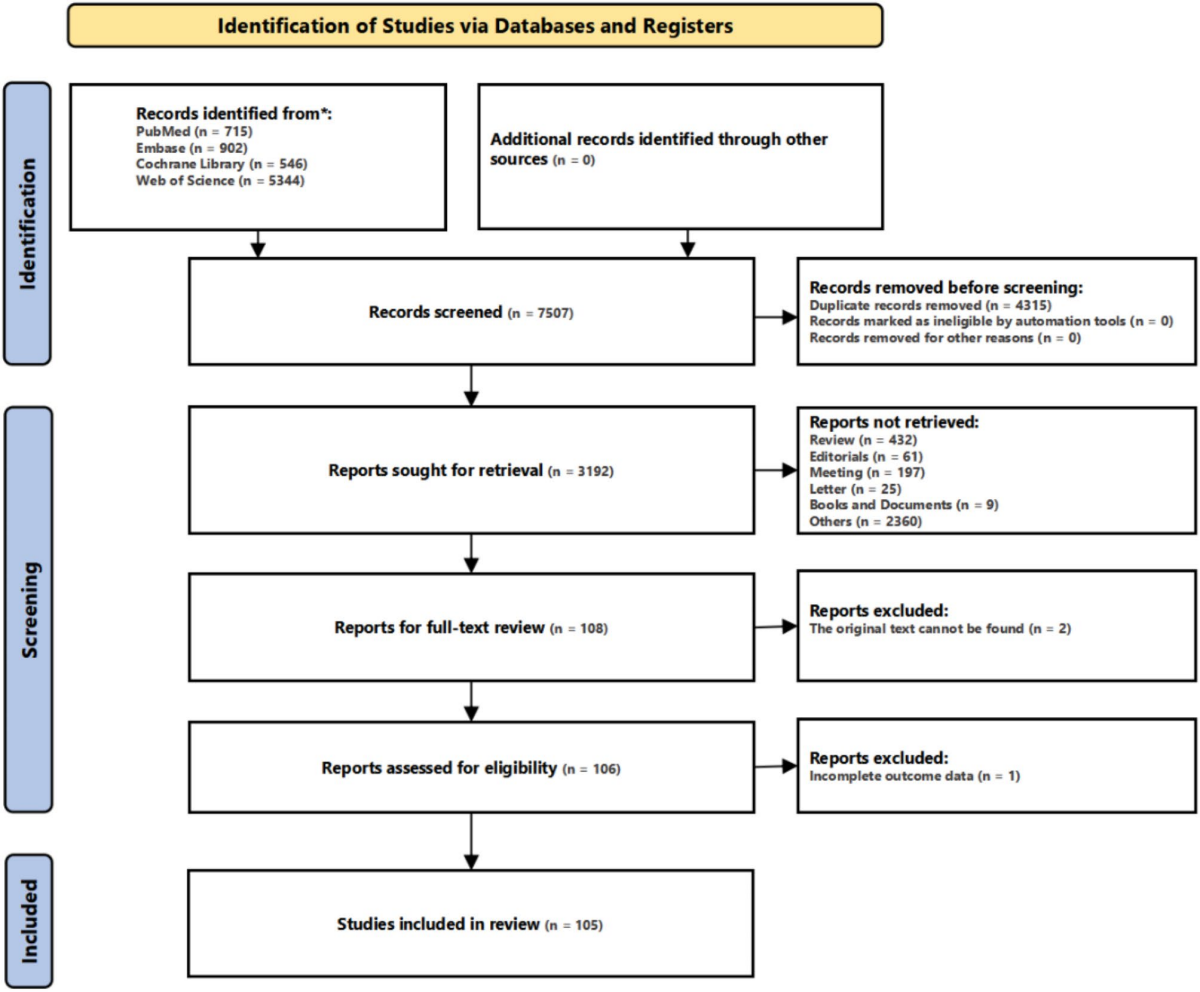
PRISMA flowchart for the screening and selection of included studies

A total of 7,507 records were identified through database searches, including 715 from PubMed, 902 from Embase, 546 from the Cochrane Library, and 5,344 from Web of Science. After the removal of 4,315 duplicate records, 3,192 records remained for screening. Following the title and abstract screening phase, 108 studies were selected for full-text review. Two studies were excluded as the original text could not be retrieved, and one study was excluded due to incomplete outcome data. Ultimately, 105 studies met the inclusion criteria and were included in the systematic review. The study selection process is outlined in Fig. 1.

### Study Characteristics

This systematic review included 105 studies, encompassing 8,294 children and adolescents with ACL injuries. Most of the studies (102, 97.14%) were single-center, with only 3 (2.86%) conducted as multicenter studies. Participant ages varied widely, with the mean age between 12 and 16 years in most studies. In terms of evidence levels, three studies (2.86%) were classified as Level II, 25 studies (23.81%) as Level III, and the majority, 77 studies (73.33%), were classified as Level IV evidence. Follow-up durations ranged from 3 to 216 months, offering insights into short-term and long-term outcomes.

### Quality Assessment

Non-comparative studies scored between 8 and 12 out of a maximum MINORS score of 16, while comparative studies achieved scores ranging from 8 to 18 out of a maximum MINORS score of 24. The assessment revealed significant variability in methodological rigor. Strengths were noted in clearly stated aims and adequate follow-up periods. However, several limitations were identified, particularly in the unbiased assessment of study endpoints and the prospective calculation of sample sizes. Detailed results of the quality assessment are provided in **Supplementary File 1**.

### Knee Instability

The occurrence rates of knee instability varied significantly across different treatments. Among the studies reporting outcomes of conservative treatments, knee instability rates ranged from 35.85–100% [5, 13, 14]. In contrast, studies evaluating the outcomes of transphyseal ACL reconstruction reported substantially lower rates of knee instability, which ranged from 0.00–7.41% [15–22].

### Secondary Damage To the Meniscus And/or Cartilage

Several studies have reported the incidence of secondary damage to the meniscus and/or cartilage in children and adolescents with ACL injuries. A prospective MRI study by Harvard et al. [23] reported that among 28 patients treated conservatively, the incidence of meniscal injuries was 28.5%, with a 3.6% incidence of new meniscal and cartilage injuries observed during two MRI evaluations. Similarly, a prospective cohort study with a 2-year follow-up involving 46 skeletally immature children found that 78% of the participants remained ACL deficient, yet knee function was satisfactory overall. However, new meniscal injuries occurred in 17% of cases over the follow-up period [24].

In contrast, surgical management demonstrated a reduced risk of meniscal damage. John et al. [25] observed that among 38 patients who initially received non-surgical treatment, 27 had symptomatic meniscal tears, whereas only one patient who underwent ACL reconstruction had a symptomatic meniscal tear. William et al. [26], studying 13 adolescents with open physes who delayed ACL reconstruction until skeletal maturity found no evidence that delaying surgery increased the risk of additional knee injuries. Julien et al. [27] conducted a retrospective study of 56 patients with open physes and found that the delayed reconstruction group experienced a significantly higher rate of medial meniscal tears (41%) compared to the early reconstruction group (16%). Similarly, Guillaume et al. [28] found that in pediatric patients with ACL injuries, those who underwent treatment more than 150 days after injury exhibited a higher incidence of medial meniscal tears (53.5%) compared to those treated within 150 days (37.8%). In addition, four studies reported the results of meniscal injury after transphyseal ACL reconstruction. The occurrence rate fluctuates between 5.26–15.38% [29–32]. Three studies reported the results of meniscal injury after all-epiphyseal ACL reconstruction, and the occurrence rate is 2.91%, 9.77%, and 18.52% [33–35]. One study reported the results of meniscal injury after physeal-sparing ACL reconstruction. The occurrence rate is 5.91% [36].

### Radiological Signs of Degenerative Changes

Radiological signs of degenerative changes in the knee joint were reported in both conservative and surgical treatment groups. Among studies evaluating conservative treatments, two studies reported high occurrence rates of degenerative changes, reported at 43.48% and 61.11% [37, 38]. These findings underscore the long-term risks associated with non-surgical management of ACL injuries in children and adolescents. In contrast, eight studies assessed radiological signs of degenerative changes following transphyseal ACL reconstruction. The reported occurrence rates in these studies ranged from 0 to 4.00%, indicating a significantly lower risk of degenerative changes compared to conservative treatment [19, 21, 39–44].

### Growth Plate Problems or Leg-Length Discrepancy

Premature physeal closures and leg-length discrepancies are important complications associated with ACL reconstruction in children and adolescents with open physes. Eight studies investigated premature physeal closures or growth plate problems following transphyseal ACL reconstruction, with reported occurrence rates ranging from 0–13.79% [19, 30, 32, 44–48]. Similarly, three studies examined these complications after all-epiphyseal ACL reconstruction, with reported rates of 0%, 0%, and 11.11% [33, 49, 50]. No premature physeal closure or growth plate problems were reported in studies evaluating partial transphyseal or physeal-sparing ACL reconstruction [36, 51]. For studies reporting the occurrence of leg-length discrepancies following transphyseal ACL reconstruction, the rates ranged from 0–6.25% [13, 17, 21, 22, 29, 32, 39–42, 46, 47, 52–56]. No cases of leg-length discrepancies were identified in studies focusing on partial transphyseal ACL reconstruction [54, 57]. Four studies assessed this complication in physeal-sparing ACL reconstruction, with reported rates ranging from 0–3.85% [36, 49, 58, 59]. Conversely, five studies on all-epiphyseal ACL reconstruction demonstrated a wider range of occurrence rates, fluctuating from 0–33.33% [49, 50, 60–62].

### Graft Failures and Repeat Surgery

The occurrence rates of graft rupture or failures varied across treatment modalities. For transphyseal or partial transphyseal ACL reconstruction, graft rupture rates ranged widely from 0–41.18% [22, 30–32, 55, 56, 63–65]. Similarly, graft rupture rates following all-epiphyseal ACL reconstruction fluctuated between 0% and 33.33%. Regarding partial transphyseal ACL reconstruction, two studies reported graft rupture rates of 5.56% and 16.70% [65, 66].

The rates of repeat surgery also demonstrated variability. For transphyseal ACL reconstruction, repeat surgery rates ranged from 3.12–37.93% [18, 19, 21, 30, 40, 52, 55, 63, 65, 67, 68]. Studies focusing on physeal-sparing ACL reconstruction reported that repeat surgery rates were 4.55%, 3.57%, and 28.57% [51, 57, 59]. For all-epiphyseal ACL reconstruction, two studies reported repeat surgery rates of 14.29% and 16.67% [50, 68]. Finally, partial transphyseal ACL reconstruction demonstrated 10.61% and 33.33% repeat surgery rates in two studies [65, 68].

### Return To Sport

**Fig. 2:**
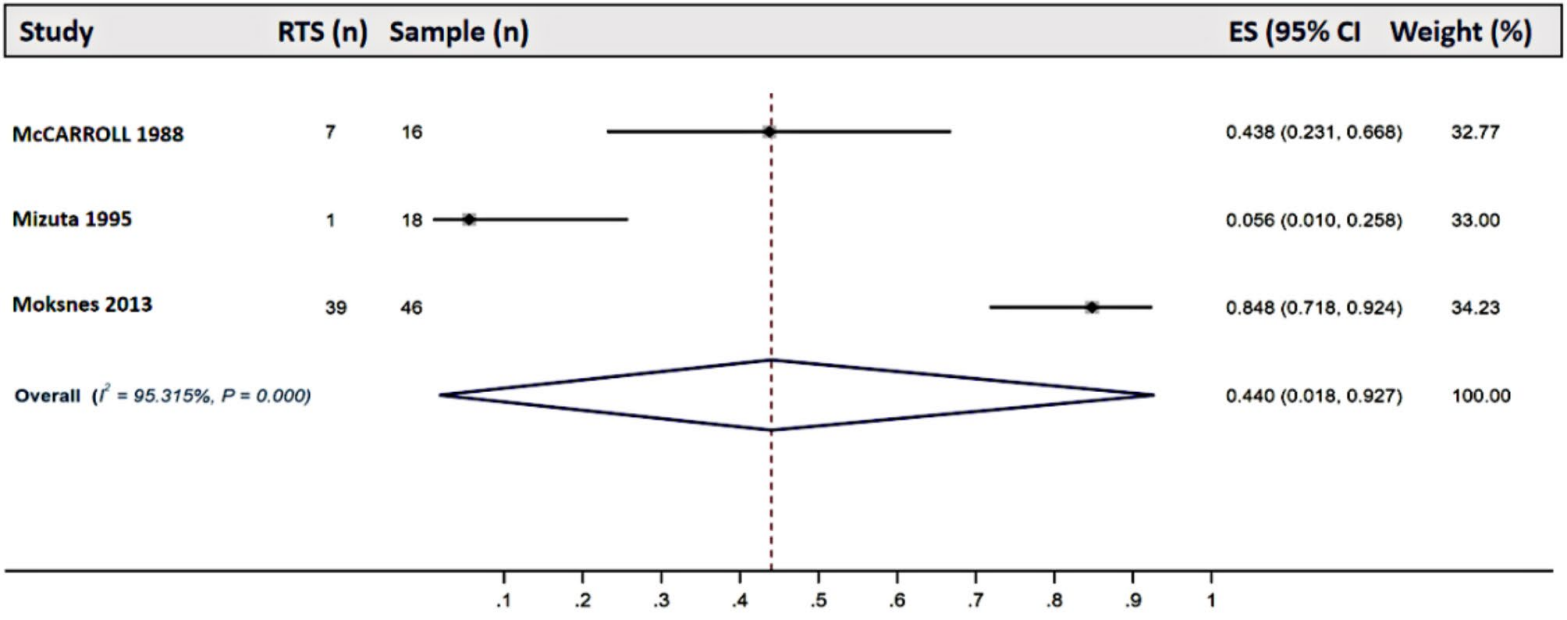
Forest plots of the pooled return-to-sport rate of studies reporting the results of conservative treatments. Abbreviations: RTS: return to sport; ES: Effect size; CI: confidence interval

**Fig. 3:**
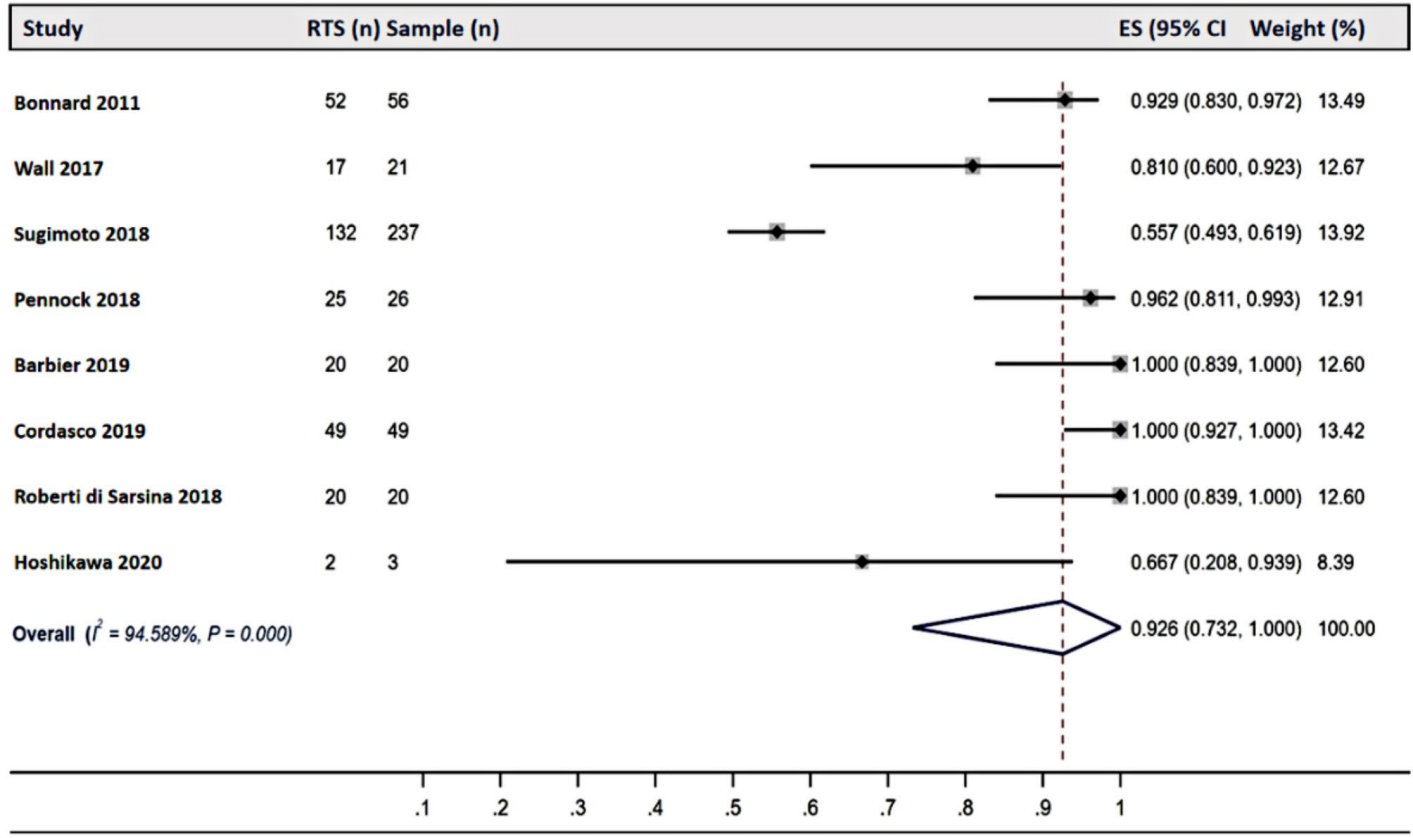
Forest plots of the pooled return-to-sport rate of studies reporting physeal-sparing anterior cruciate ligament reconstruction results. Abbreviations: RTS: return to sport; ES: Effect size; CI: confidence interval

**Fig. 4:**
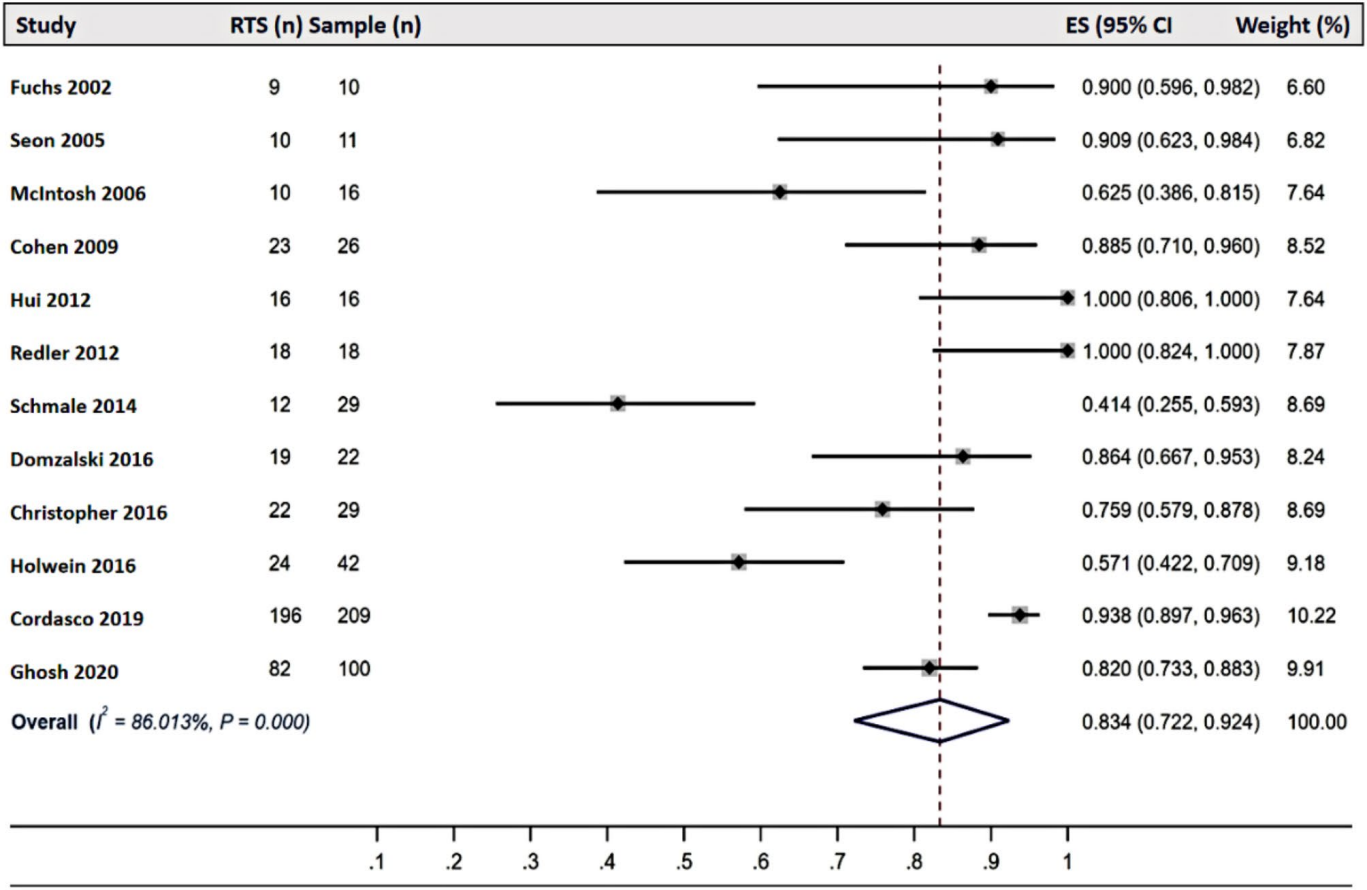
Forest plots of the pooled return-to-sport rate of studies reporting the results of transphyseal anterior cruciate ligament reconstruction. Abbreviations: RTS: return to sport; ES: Effect size; CI: confidence interval

The rate of RTS varied significantly across different interventions. Among studies reporting outcomes of conservative treatments, RTS rates ranged from 5.56 to 84.78%, with a pooled RTS rate of 44.00% (95% CI: 0.02–0.93) [24, 37, 69](Fig. 2). For physeal-sparing ACL reconstruction, RTS rates ranged from 55.70 to 100.00%, with a pooled RTS rate of 92.60% (95% CI: 0.73–1.00) [33, 36, 51, 54, 60, 61, 68, 70] (Fig. 3). Similarly, studies on transphyseal ACL reconstruction reported RTS rates ranging from 41.38 to 100.00%, with a pooled RTS rate of 83.40% (95% CI: 0.72–0.92) [15, 16, 21, 39, 44, 46–48, 53, 56, 67, 68](Fig. 4).

## Discussion

The incidence of ACL injuries in children and adolescents has increased significantly over the past few decades, leading to ongoing debate and controversy regarding the optimal treatment strategy [71]. While surgical reconstruction using autografts or allografts has been the standard treatment for ACL deficiency in adults, children and adolescent populations present unique challenges due to open physes and the potential risk of growth disturbances [72]. This systematic review comprehensively evaluates management strategies for ACL injuries in children and adolescents, focusing on clinical outcomes, complications, and RTS rates across different treatment modalities. The findings revealed notable differences among treatments. Conservative treatments were associated with elevated rates of knee instability, significant risks of secondary meniscal and cartilage damage, and a higher incidence of long-term degenerative changes. Furthermore, RTS rates following conservative treatments were relatively low. In contrast, ACL reconstruction demonstrated superior outcomes, including significantly reduced rates of knee instability, secondary damage, and degenerative changes, coupled with higher pooled RTS rates. However, ACL reconstruction is associated with the risk of potential graft rupture and the need for repeat surgeries. Early surgical interventions were more effective than conservative treatments in restoring knee stability, reducing complications, and improving long-term outcomes.

A retrospective cohort study by Kessler et al. [73] demonstrated that surgical ACL reconstruction lowers the risk of secondary meniscal tears and mitigates the negative effects of osteoarthritis (OA) compared to conservative treatment. However, recent evidence suggests that surgical management may also increase the risk of early-onset OA [74]. A meta-analysis by Cuzzolin et al. [75] revealed that surgical treatment for ACL injuries offers superior clinical outcomes, including improved knee joint function and a lower incidence of secondary meniscectomies, compared to conservative management. Similarly, a systematic review and meta-analysis by James et al. [8] showed that delaying ACL reconstruction for more than 12 weeks significantly increases the risk of meniscal damage and irreparable meniscal tears. Despite this, early and delayed surgical interventions were found to provide satisfactory knee stability. In contrast, non-surgical treatments were associated with higher rates of residual knee instability, an increased risk of meniscal tears, and lower return-to-sport rates.

Growth disturbances, such as leg shortening or angular deformities, remain critical in early surgical interventions for children and adolescents. A systematic review by Mohtadi et al. [76] concluded that current evidence is insufficient to recommend either early or delayed ACL reconstruction for skeletally immature individuals. However, a review by Dunn et al. [77] found that early ACL reconstruction reduced knee instability and increased return-to-sport rates without negatively affecting growth plates or causing growth disturbances. Similarly, Longo et al. [78] reported low rates of growth disturbances following ACL reconstruction in skeletally immature individuals. Nonetheless, the available evidence remains limited, and no single surgical technique has been shown to minimize growth disturbances or produce superior clinical outcomes consistently.

**Fig. 5:**
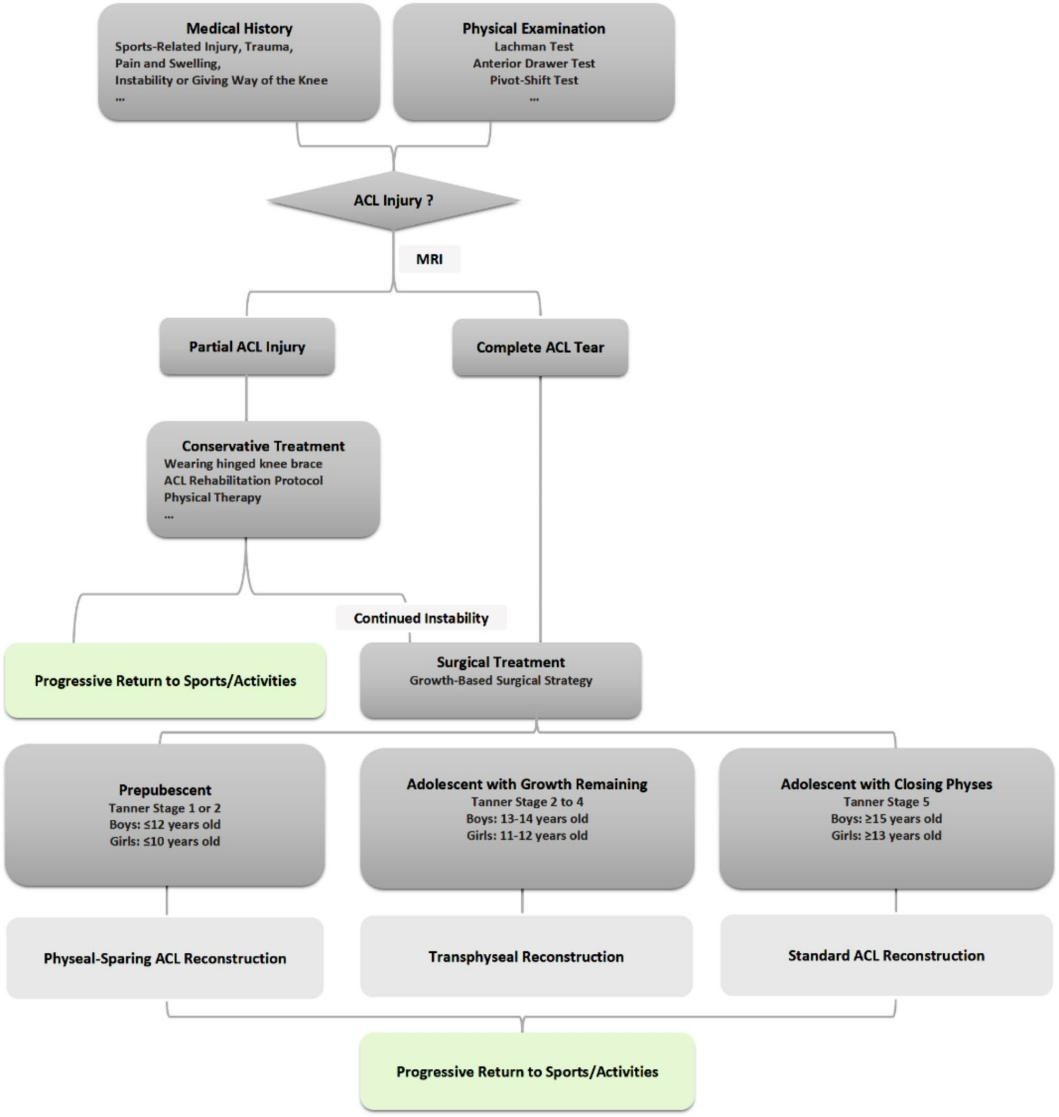
Management of anterior cruciate ligament injuries in children and adolescents. Abbreviations: ACL: Anterior Cruciate Ligament; MRI: Magnetic Resonance Imaging

Multiple surgical techniques have been developed for pediatric ACL reconstruction, including physeal-sparing techniques, epiphyseal techniques, partial transphyseal techniques, and bridge-augmentation techniques, all aimed at restoring knee stability and kinematics [31, 79, 80]. Among these, physeal-sparing techniques demonstrate a favorable safety profile and pose minimal risk of growth disturbances, particularly in prepubescent children at Tanner stages 1 and 2. One example is the “over-the-top” physeal-sparing technique, which combines intra- and extra-articular reconstruction using an iliotibial (IT) band autograft positioned “over-the-top” on the femur, effectively minimizing disruption to the physeal region [81]. Another physeal-sparing option is all-epiphyseal ACL reconstruction, which involves graft fixation entirely within the epiphysis, avoiding the physis, and using either the quadriceps tendon or hamstring autograft [82]. A narrative review by Wong et al. [83] compared the outcomes of the “over-the-top” and all-epiphyseal techniques, reporting that the all-epiphyseal group showed a higher incidence of overgrowth, while the “over-the-top” group demonstrated a higher incidence of angular deformities. However, the risk of graft rerupture was similar between the two techniques. Transphyseal ACL reconstruction, a widely used technique in adolescents, involves creating steep femoral tunnels, also referred to as non-anatomical tunnels, which can protect Ranvier’s zone, a critical area associated with growth disturbances in the skeletal system [84]. Despite this, the rotational stability and ability to prevent OA may be slightly inferior compared to anatomical techniques [85]. Anatomical techniques are more commonly recommended for adolescents with closed physes. A systematic review by Pierce et al. [9] found no significant differences in growth disturbances or graft survival between transphyseal and physeal-sparing techniques in skeletally immature individuals, supporting the use of either approach. Similarly, a systematic review by Kaeding et al. [86] highlighted that both physeal-sparing and transphyseal techniques yield excellent clinical outcomes with a very low incidence of growth complications in patients at Tanner stages II and III. Notably, Tanner stage I patients achieved optimal outcomes with physeal-sparing techniques. These findings support expanding the indications for transphyseal reconstruction from Tanner stage IV patients to include Tanner stage II and III patients. This body of evidence provides valuable guidance for clinicians in selecting the most appropriate surgical technique based on the patient’s Tanner stage, growth potential, and individual anatomical considerations (Fig. 5). Considering the elevated rates of revision surgeries among young, active individuals returning to pivoting sports, careful management and tailored surgical approaches are essential [87]. The variability in graft failure and repeat surgery rates across different surgical techniques for ACL reconstruction in skeletally immature individuals highlights the complexity of managing these cases. Research indicates that multiple ACL reconstruction revisions can yield satisfactory clinical outcomes and maintain knee stability with a low risk of re-rupture. However, the chances of returning to pre-injury sports activity levels remain limited [88].

Our study has several limitations that should be acknowledged. By restricting our review to peer-reviewed, published studies, we may have excluded valuable data from gray literature. Additionally, the majority of the included studies were of lower-level evidence (LOE III-IV) due to the limited availability of high-quality randomized controlled trials in this field. This reliance on observational studies may affect the reliability and generalizability of our findings. In the “*Return to Sport*” section, we used a single-group rate approach to meta-analysis. While this method effectively summarizes descriptive outcomes, it does not provide comparative results with statistical significance, limiting the use of bias assessments and sensitivity analysis. Furthermore, although high heterogeneity was observed among the included studies, we did not explore the potential sources of heterogeneity in detail, which warrants caution when interpreting these findings. Another limitation relates to the relatively small sample sizes of some included case series, which could impact the statistical power and consistency of the reported results. Larger-scale studies may yield outcomes that differ from our findings, potentially influencing the robustness of our conclusions. While our study evaluated key outcomes such as knee instability, secondary meniscal and cartilage injuries, growth disturbances, and RTS rates, other important patient-centered outcomes, such as quality of life and pain levels, were not comprehensively reviewed due to limitations in the available data. Moreover, our study did not include research focusing on adjunctive procedures, such as anterolateral ligament reconstruction. The omission of this aspect limits the comprehensiveness of our findings. Finally, our review included only studies published in English, potentially excluding relevant research in other languages. This language restriction introduces the possibility of selection bias and may have limited the comprehensiveness of our analysis.

## Conclusion

Conservative treatments for ACL injuries in children and adolescents are associated with elevated rates of knee instability, secondary meniscal and cartilage damage, and long-term degenerative changes. In contrast, surgical interventions consistently demonstrate superior outcomes, including effective restoration of knee stability, reduced complications, and higher RTS rates. Nevertheless, the potential risks of graft rupture, repeat surgeries, and growth disturbances underscore the critical need for a personalized approach, with surgical techniques carefully tailored to the patient’s growth stage and anatomical characteristics. Although early surgical intervention shows significant promise in improving functional outcomes and reducing complications compared to conservative treatments, further high-quality randomized controlled trials are essential to validate these findings and establish evidence-based guidelines for optimal clinical decision-making.

## Electronic Supplementary Material

Below is the link to the electronic supplementary material.

Supplementary Material 1

## Data Availability

The datasets used and/or analysed during the research are available from the corresponding author upon reasonable request.

## Abbreviations

ACL: Anterior cruciate ligament
RTS: Return to sport
HT: Hamstring tendon
ACLR: Anterior cruciate ligament reconstruction
PRISMA: Preferred Reporting Items for Systematic Reviews and Meta-analysis
MINORS: Methodological Index for Nonrandomized Studies
KOA: Knee osteoarthritis
BTB: Bone-patellar tendon-bone
