# Supplementary material for: Management of Anterior Cruciate Ligament Injuries in Children and Adolescents: A Systematic Review
Source: Sports Med Open. 2025 Apr 23;11:40. doi: 10.1186/s40798-025-00844-7 (PMC12014893; doi:10.1186/s40798-025-00844-7)
Supplement: Supplementary file 1 — Supplementary Material 1 [file 40798_2025_844_MOESM1_ESM.pdf]

Supplementary File 1

Methodological Index for Non-Randomized Studies (MINORS)													
Study	Q1	Q2	Q3	Q4	Q5	Q6	Q7	Q8	Q9	Q10	Q11	Q12	Total
Lipscomb AB 1986	2	1	2	1	0	2	1	0					9/16
JOHN R. McCARROLL 1988	2	2	2	2	0	2	2	0	1	0	1	1	15/24
Ben K. Graf 1992	2	0	2	0	1	2	0	0	0	0	0	1	8/24
Michelle Andrews 1994	2	0	2	1	1	2	0	0					8/16
John R. McCarroll 1994	2	2	2	1	1	2	0	0	1	0	0	1	12/24
H Mizuta 1995	2	0	2	1	0	2	1	0					8/16
Janarv Per-Mats 1996	2	0	2	1	1	2	1	0	1	0	1	1	12/24
Pressman A. E 1997	2	2	2	1	1	2	1	0	1	0	1	2	15/24
Lyle J. Micheli 1999	2	0	2	1	1	2	2	0					10/16
Eric R. Aronowitz 2000	2	0	2	1	1	2	2	0					10/16
P. M. Aichroth 2000	2	0	2	1	1	2	2	0	2	1	1	1	15/24
Pete H. Edwards 2001	2	0	2	1	1	2	2	0					10/16
Robin Fuchs 2002	2	0	2	2	2	2	1	0					11/16
Mininder S. Kocher 2002	2	0	2	2	2	2	1	0					11/16
Peter J. Millett 2002	2	0	2	1	1	2	1	0					9/16
Vincenzo Guzzanti 2003	2	1	2	2	2	2	1	0					12/16
G. William Woods 2004	2	1	2	1	1	2	1	0	2	1	1	1	15/24
K. Donald Shelbourne 2004	2	1	2	1	1	2	2	0					11/16
Mininder S. Kocher 2005	2	1	2	1	1	2	1	0					10/16
Jong Keun Seon 2005	2	1	2	1	1	2	1	0					10/16
J. Richard Steadman 2006	2	1	2	1	1	2	0	0					9/16
H.M. Gaulrapp 2006	2	1	2	1	1	2	1	0					10/16
F. Gebhard 2006	2	1	2	1	1	2	2	0	2	1	1	0	15/24
Amy L. McIntosh 2006	2	1	2	1	1	2	1	0					10/16
Mininder S. Kocher 2007	2	1	2	1	1	2	1	0					10/16
A. D. Liddle 2008	2	1	2	1	1	2	1	0					10/16
Wudbhav N. Sankar 2008	2	1	2	1	1	2	1	0					10/16
Moises Cohen 2009	2	2	2	1	1	2	2	0					12/16
Julien Henry 2009	2	1	2	1	1	2	1	0	2	1	1	1	15/24
Nikolaus A. Streich 2010	2	1	2	1	1	2	1	0	2	1	1	1	15/24
Aurelien Courvoisier 2010	2	0	2	1	1	2	1	0					9/16
Pantelis Nikolaou 2011	2	1	2	1	1	2	1	0					10/16
J. Todd R. Lawrence 2011	2	1	2	1	1	2	2	0					11/16
Walter P. Samora 2011	2	2	2	1	1	2	2	0					12/16
C. Bonnard 2011	2	1	2	1	1	2	1	0					10/16
Guillaume D. Dumont 2012	2	1	2	1	1	2	1	0					10/16
Catherine Hui 2012	2	1	2	1	1	2	1	0					10/16

Supplementary File 3

Sung-Jae Kim 2012	2	1	2	1	1	2	1	0					10/16
Lauren H. Redler 2012	2	1	2	2	2	2	1	0					12/16
Sujit Kumar 2013	2	1	2	2	1	2	1	0					11/16
Håvard Moksnes 2013	2	1	2	2	1	2	1	0					11/16
Håvard Moksnes 2013	2	1	2	2	1	2	1	0					11/16
Martin Goddard 2013	2	2	2	2	1	2	1	0					12/16
Sandro Kohl 2013	2	1	2	2	1	2	1	0					11/16
Xavier Cassard 2013	2	1	2	2	1	2	1	0					11/16
Glenn H. Engelman 2014	2	1	2	2	1	2	1	0	2	1	1	1	16/24
Marco Kawamura Demange 2014	2	1	2	2	1	2	1	0					11/16
Gregory A. Schmale 2014	2	1	2	2	1	2	1	0					11/16
G. Lemaitre 2014	2	1	2	1	1	2	1	0					10/16
Peter P. Koch 2014	2	1	2	2	1	2	1	0					11/16
Danyal H. Nawabi 2014	2	1	2	2	1	2	1	0					11/16
Justin T. Newman 2014	2	2	2	2	1	2	1	0	2	1	1	1	17/24
Rafael Calvo 2014	2	1	2	2	1	2	1	0					11/16
Duncan Reid 2015	2	2	2	2	1	2	1	0					12/16
Aristides I Cruz Jr 2015	2	1	2	1	1	2	1	0					10/16
S. Clifton Willimon 2015	2	2	2	2	1	2	1	0					12/16
Allen F. Anderson 2015	2	2	2	2	1	2	1	0	2	1	1	2	18/24
Marcin Domzalski 2015	2	2	2	2	1	2	1	0					12/16
Luca Dei Giudici 2016	2	2	2	2	1	2	1	0					12/16
Christopher M Larson 2016	2	2	2	2	1	2	1	0					12/16
C. Holwein 2016	2	1	2	2	1	2	1	0					11/16
Giacomo Placella 2016	2	1	2	2	1	2	1	0					11/16
Francesco Falciglia 2016	2	1	2	1	1	2	1	0					10/16
Eric J. Wall 2017	2	1	2	2	1	2	1	0					11/16
Anya Madelaine 2018	2	2	2	1	1	2	1	0					11/16
Dai Sugimoto 2018	2	2	2	2	1	2	1	0	2	1	1	1	17/24
Mininder S. Kocher 2018	2	1	2	1	1	2	1	0					10/16
Julie P. Burland 2018	2	2	2	2	1	2	1	0					12/16
Loïc Geffroy 2018	2	1	2	1	1	2	1	0					10/16
Andrew T. Pennock 2018	2	1	2	1	1	2	1	0					10/16
Adam J. Tagliero 2018	2	2	2	1	1	2	1	0					11/16
Alex G. Dukas 2018	2	1	2	2	1	2	1	0					11/16
Tommaso Roberti di Sarsina 2018	2	2	2	2	1	2	1	0					12/16
Emma L. Heath 2018	2	2	2	2	1	2	1	0					12/16
Sheena C. Ranade 2018	2	1	2	2	1	2	1	0					11/16
Neeraj M. Patel 2018	2	1	2	2	1	2	1	0					11/16
D. Barbier 2018	2	1	2	1	1	2	1	0					10/16

Supplementary File 3

Ahmad F. Bayomy 2018	2	2	2	2	1	2	1	0					12/16
Nirav K. Pandya 2019	2	1	2	1	1	2	1	0					10/16
Crystal A. Perkins 2019	2	1	2	2	1	2	1	0					11/16
Frank A. Cordasco 2019	2	1	2	1	1	2	1	0					10/16
Andrew T. Pennock 2019	2	2	2	2	1	2	1	0	2	1	1	2	18/24
Mohammad Razi 2019	2	1	2	1	1	2	1	0	2	1	1	1	15/24
Regina O. Kostyun 2019	2	2	2	2	1	2	1	0					12/16
Stephen Mathew 2019	2	2	2	2	1	2	1	0					12/16
Alexia G. Gagliardi 2019	2	1	2	2	1	2	1	0					11/16
Christian Konrads 2020	2	2	2	2	1	2	1	0	2	1	1	1	17/16
Atsuto Hoshikawa 2020	2	1	2	2	1	2	1	0					11/16
Lilah Fones 2020	2	2	2	2	1	2	1	0					12/16
Erica L. Holland 2020	2	2	2	2	1	2	1	0	2	1	1	1	17/24
Joseph P. Hannon 2020	2	1	2	2	1	2	1	0					11/16
Koushik Ghosh 2020	2	1	2	1	1	2	1	0					10/16
Adam Weaver 2021	2	2	2	2	1	2	1	0	2	1	1	2	18/24
Natalie H. Vaughn 2021	2	1	2	1	1	2	1	0					10/16
Alexander Zimmerer 2021	2	1	2	2	1	2	1	0	2	1	1	1	16/24
Alexandra H. Aitchison 2021	2	1	2	1	1	2	1	0					10/16
Conor J. Kilkenny 2022	2	2	2	1	1	2	1	0	2	1	1	1	16/24
Jordan R Nester 2022	2	1	2	1	1	2	1	0					10/16
Liang Zhang 2022	2	1	2	2	1	2	1	0	2	1	1	1	16/24
Joseph M. Brutico 2022	2	1	2	2	1	2	1	0	2	1	1	1	16/24
Alan G. Shamrock 2022	2	2	2	2	1	2	1	0					12/16
Sachin Allahabadi 2022	2	2	2	2	1	2	1	0	2	1	1	1	17/24
Constant Foissey 2022	2	2	2	2	1	2	1	0					12/16
Katharine F. Hollnagel 2023	2	2	2	2	1	2	1	0	2	1	1	2	18/24
Daniel W. Green 2023	2	1	2	2	1	2	1	0	2	1	1	1	16/24

Q1. Clearly stated aim; Q2. Inclusion of consecutive patients; Q3. Prospective collection of data; Q4. Endpoints appropriate to the aim of the study; Q5. Unbiased assessment of the study endpoint; Q6. Follow-up period appropriate to the aim of the study; Q7. Loss to follow up $< 5\%$; Q8. Prospective calculation of the study size; Q9. Adequate control group; Q10. Contemporary groups; Q11. Baseline equivalence of groups; Q12. Adequate statistical analyses.
